# Evaluation of Indigenous Olive Biocontrol Rhizobacteria as Protectants against Drought and Salt Stress

**DOI:** 10.3390/microorganisms9061209

**Published:** 2021-06-03

**Authors:** Nuria Montes-Osuna, Carmen Gómez-Lama Cabanás, Antonio Valverde-Corredor, Garikoitz Legarda, Pilar Prieto, Jesús Mercado-Blanco

**Affiliations:** 1 Departamento de Protección de Cultivos, Instituto de Agricultura Sostenible, Agencia Estatal Consejo Superior de Investigaciones Científicas (CSIC), Avenida Menéndez Pidal s/n, Campus “Alameda del Obispo”, 14004 Córdoba, Spain; nuriamontes@ias.csic.es (N.M.-O.); cgomezlama@ias.csic.es (C.G.-L.C.); valverde@ias.csic.es (A.V.-C.); 2 Unidad de Bioinformática, Sistemas Genómicos S.L., Ronda G. Marconi, 6, Parque Tecnológico de Valencia, 46980 Paterna, Spain; garikoitz.legarda@sistemasgenomicos.com; 3 Departamento de Mejora Genética Vegetal, Instituto de Agricultura Sostenible, Agencia Estatal Consejo Superior de Investigaciones Científicas (CSIC), Avenida Menéndez Pidal s/n, Campus “Alameda del Obispo”, 14004 Córdoba, Spain; pilar.prieto@ias.csic.es

**Keywords:** 1-aminocyclopropane-1-carboxylic acid (ACC) deaminase (ACD), chlorophyll (Chl), flavonoid (Flv), proline, *Pseudomonas* sp. PICF6, *Pseudomonas simiae* PICF7, stem water potential (Ψ), stomatal conductance (gs)

## Abstract

Stress caused by drought and salinity may compromise growth and productivity of olive (*Olea europaea* L.) tree crops. Several studies have reported the use of beneficial rhizobacteria to alleviate symptoms produced by these stresses, which is attributed in some cases to the activity of 1-aminocyclopropane-1-carboxylic acid deaminase (ACD). A collection of beneficial olive rhizobacteria was in vitro screened for ACD activity. *Pseudomonas* sp. PICF6 displayed this phenotype and sequencing of its genome confirmed the presence of an *acdS* gene. In contrast, the well-known root endophyte and biocontrol agent *Pseudomonas simiae* PICF7 was defective in ACD activity, even though the presence of an ACD-coding gene was earlier predicted in its genome. In this study, an unidentified deaminase was confirmed instead. Greenhouse experiments with olive ‘Picual’ plants inoculated either with PICF6 or PICF7, or co-inoculated with both strains, and subjected to drought or salt stress were carried out. Several physiological and biochemical parameters increased in stressed plants (i.e., stomatal conductance and flavonoids content), regardless of whether or not they were previously bacterized. Results showed that neither PICF6 (ACD positive) nor PICF7 (ACD negative) lessened the negative effects caused by the abiotic stresses tested, at least under our experimental conditions.

## 1. Introduction

Olive (*Olea europaea* L. subsp. *europaea* var. *europaea*) is probably the most emblematic tree crop of the Mediterranean Basin, with a huge social, economic, and environmental importance [1]. It is well adapted to the climatic conditions usually found in this region, characterized by high temperatures and low rainfall during the summer season. However, recent studies on the effects of climate change have projected an increase in temperatures in this geographical area which could aggravate events of severe drought thereby threatening olive production [2,3,4]. Moreover, this scenario may become more serious in coastal areas where the use of fresh water in agriculture could be restricted [5]. As a result, the use of saline water or even reclaimed water, which in some cases may contain significant amounts of salt, would be necessary [6]. Salt stress affects millions of hectares around the world, compromising cultivated areas that produce a third of the world’s food. Drought affects a high percentage of the global surface (30%) in arid and semiarid regions [7].

High rates of evaporation, insufficient leaching and the use of salinized water for irrigation are increasing problems in olive cultivation [8,9]. Despite olive trees are able to tolerate water scarcity and have developed physiological adaptations to salt stress, they can experience stress when these conditions become more severe or prolonged [10,11,12]. Drought stress directly affects olive plants metabolism, reducing productivity due to stunted growth and lower biomass production [13]. Nevertheless, this assumption is not confirmed in olive trees subjected to salt stress. Data from diverse studies are indeed controversial, and while it is generally acknowledged that high salt concentrations diminish olive yield [14,15], other reports showed the opposite conclusion [16,17].

Plants have evolved different defense stratagems to alleviate the negative effects caused by drought and/or salt stress. Overall, these strategies are based on three types of responses: (a) mechanisms aimed to avoid the loss of water (i.e., osmotic homeostasis), (b) mechanisms based on cellular component protection (i.e., qualitative and quantitative changes of pigments) and, finally, (c) mechanisms to repair oxidative damage or detoxification (i.e., antioxidant defense system) [18,19]. In this sense, changes in stem water potential (Ψ) and stomatal conductance (gs) are responses developed by the plant to maintain its homeostasis [20]. The decrease in chlorophyll (Chl) content directly affects plant development in stressed plants [21], while the accumulation of phenolic compounds such as flavonoids (Flv) or osmolytes like proline are excellent indicators of plant stress [22].

Over the last few years, the use of plant growth-promoting microorganisms has been proposed as an approach to alleviate or minimize effects caused by different types of abiotic stress. Recently, Kumar and co-workers [9] have extensively reviewed the mechanisms of salt stress tolerance in different plants, including olive, previously inoculated with plant growth promoting rhizobacteria (PGPR). These mechanisms include the production of extracellular polymeric substances, nitrogen fixation, phytohormone biosynthesis (i.e., indole-3-acetic acid), production of antioxidants, or the activity of 1-aminocyclopropane-1-carboxylic acid (ACC) deaminase (ACD) [9,23]. This PGPR-produced enzyme seems to play an important role in increasing plant’s tolerance to stress, in addition to plant growth promotion [24]. ACD catalyses the conversion of ACC, the immediate precursor in the ethylene (ET) biosynthetic pathway in higher plants [25,26]. Ethylene is an essential phytohormone involved in several physiological processes in plants. This gaseous hormone is involved in a large number of processes such as seed germination, leaf and flower senescence, root hair development and elongation, degreening and fruit ripening, or production of volatiles responsible for fruit aroma [27,28,29,30,31]. In addition, ET also regulates plant responses to biotic and abiotic stresses. Drought and salinity stresses induce ET biosynthesis, causing an explosive increase in its concentration and inducing negative effects in plants [32,33]. ACD-producing microorganisms can degrade ACC into α-ketobutyrate and ammonia, thereby decreasing ET levels in plants [25,26]. Win and co-workers [34] have reported that *Pseudomonas* OFT2 and *Pseudomonas* OFT5, both ACD-expressing endophytes, ameliorated the effects caused by salinity in tomato plants. Similarly, inoculation of wheat plants with ACD-producing *Pseudomonas* spp. strains had a positive impact on different growth parameters (i.e., seed vigor, length and dry weigh) when cultivated under salinity conditions [35]. Also, wheat plants inoculated with *Variovorax paradoxus* RAA3 or a consortium of different *Pseudomonas* spp. and *Ochrobactrum anthropi* (all of them showing high level of ACD activity) improved their antioxidant properties compared to non-inoculated plants under water stress [36]. However, the outcomes can be controversial. Indeed, it has also been reported that addition of ACD-producing microorganisms did not improve the altered physiological parameters in plants subjected to different types of stress, such as drought or salinity [37,38].

A collection of indigenous olive (cultivar [cv]. Picual) rhizobacteria was generated in our previous works [39,40]. Further identification and in-depth characterization allowed selecting promising strains that eventually were qualified as effective biocontrol agents (BCA) against Verticillium wilt of olive (VWO), a serious disease caused by the soil-borne fungus *Verticillium dahliae* Kleb. [1]. Among these rhizobacteria, *Pseudomonas simiae* PICF7 [41,42] and *Pseudomonas* sp. PICF6 (formerly identified as *P. fluorescens*) highlighted as good BCA against VWO [39]. While strain PICF7 has been amply characterized and showed high versatility as beneficial rhizobacteria [43,44,45,46], the available information about PICF6 is still very limited.

Further analysis of selected olive rhizobacteria, including these two BCA, as for their ability to be used as host protectants against different abiotic stresses could represent an important added value as agro-biotechnological tools. Therefore, the main objective of this study was to evaluate olive beneficial rhizobacteria as protective agents to alleviate the consequences of drought and salt stress. We aim to identify ACD producers originating from the olive rhizosphere/roots and to test the hypothesis that selected ACD producers lessen the effects of salt stress and water scarcity.

## 2. Materials and Methods

### 2.1. In Vitro Detection of ACD Activity in Selected Olive Rhizobacteria

A selection of 31 olive rhizobacteria (Supplementary Table S1) originating from olive (cv. Picual) plants purchased in different commercial nurseries located in Córdoba province (southern Spain), and previously identified and characterized in our laboratory [39,47,48], was screened as for their ability to use ACC as a sole nitrogen source (presence of ACD activity). Experiments were performed on both liquid and solid Dworkin-Foster (DF) medium [49] containing 3 mM ACC instead of (NH_4_)_2_SO_4_ as nitrogen source. This medium was prepared as described by Penrose and Glick [50]. In these assays, pH was adjusted at 7.2 (optimal value to grow the bacterial selection assayed). For determination of ACD activity in solid medium, agar (20 g/L) (Oxoid, Basingstoke, UK) was added to the DF medium. A filter-sterilized (0.2 µm membrane) ACC (≥98%, Alfa Aesar, Thermo Fisher (Kandel) GmbH, Karlsruhe, Germany) stock solution (0.1 M) was prepared in distilled water and stored at −20 °C until use. Bacteria were grown overnight in Luria Bertani liquid medium (LB, 5 g yeast extract, 10 g triptone and 5 g NaCl in 1000 mL of distilled water, 28 °C, 140 rpm). After that, ACD activity assays in solid and liquid media were performed in parallel. On the one hand, aliquots (500 µL) of each bacterial culture were transferred to a new sterile tube. Bacterial cells were harvested by centrifugation (3000× *g* rpm, 5 min), washed and resuspended in 500 µL of 10 mM sterile MgSO_4_·7H_2_O. Subsequently, 10 µL droplets of each strain were deposited on agar plates of: (i) DF medium, (ii) DF medium amended with 3 mM of ACC instead of (NH_4_)_2_SO_4_ as nitrogen source, and (iii) DF medium without nitrogen source (negative control). Plates were then incubated at 28 °C during 3 days. On the other hand, an aliquot (2% *v*/*v*) from the initial overnight culture was transferred to a new tube containing fresh DF medium that was incubated (28 °C, 140 rpm) for 24 h. Afterwards, a new aliquot (2% *v*/*v*) was transferred from this last culture into a new tube containing DF medium with 3 mM ACC (instead of (NH_4_)_2_SO_4_) and another tube with DF medium without any nitrogen source (negative control). Tubes were incubated as mentioned above. Growth on DF medium amended with ACC was taken as an indicator of positive ACD activity. Strains *Pseudomonas fluorescens* YsS6 [51] and its ACC deaminase defective mutant (YsS6 acdS−) [52], kindly provided by Dr. Franscisco X. Nascimento (Universidade NOVA de Lisboa), were included as positive and negative controls, respectively. Additionally, *Pseudomonas simiae* WCS417 [53], a nearly isogenic strain of PICF7 strain [42], was included in these assays.

### 2.2. Sequence Analysis of ACD- and Related Deaminase-Coding Genes of Pseudomonas sp. PICF6 and Pseudomonas simiae PICF7

Earlier, a gene putatively coding for an ACD was in silico identified in the genome of *P. simiae* PICF7 [41]. However, this rhizobacteria does not show ACD activity (see the Results section). In order to confirm or discard the presence of an ACD-coding gene in PICF7, the sequence of this putative gene was compared with true *acdS* and closely-related deaminases sequences (i.e., D-cysteine desulfhydrase and undefined deaminases) from 22 selected bacterial strains previously analyzed [54]. Sequences were obtained from the *Pseudomonas* genome (https://www.pseudomonas.com/, accessed on 23 February 2019) and the Kyoto Encyclopedia of Genes and Genomes (KEGG) (https://www.genome.jp/kegg/, accessed on 23 February 2019) databases. Sequences of ACD-coding and deaminase-coding genes of *Pseudomonas* sp. PICF6, that displayed ACD activity (see the Results section), were included in this analysis. All DNA sequences were aligned using Clustal 2.0.12. (https://www.ebi.ac.uk/Tools/msa/clustalo/, accessed on 23 February 2019). Phylogenetic analysis was conducted using the Maximum Likelihood method in MEGA version 7.0 software [55] and results were displayed in a dendrogram. Reliability of the inferred tree was tested by 1000 bootstrap replications.

### 2.3. Sequencing and Assembly of the Pseudomonas sp. PICF6 Genome

*Pseudomonas* sp. PICF6 DNA was obtained by using the “JETFLEX Genomic DNA Purification Kit” (Genomed, Löhne, Germany), according to the specifications of the manufacturer. The genome of strain *Pseudomonas* sp. PICF6 was sequenced following a high-throughput sequencing strategy by using an Illumina MiSeq (2015 Illumina, Inc., San Diego, CA, USA) system, paired-end technology and *de-novo* sequencing protocol implemented at Sistemas Genómicos S.L (Paterna, Valencia, Spain). The read size was 300 bp for the paired-end reads (150 bp for each R1 and R2). The quality of the raw data was checked using FASTQC tools [56]. All adaptors were removed using the Fastq mcf tool (v1.04.803) [57]. A quality filter was made with Cutadapt (v1.9.1) [58] using a quality window value of 30. Paired-end reads were merged using Flash (v1.2.11) [59]. To mask low quality bases, the assembler Megahit (1.0.3–29-g707d683) [60] was used. Several k-mers (sizes from 15 to 99) were employed. Glimmer3 [61,62] was used for gene detection and ORFs were annotated with Blast 0.2.2.30+ [63] with an E-value cutoff of 1e^−3^ against the latest version (UniProtKB/Swiss-Prot Release 2015_08) of the Uniprot Swissprot protein curated database for bacteria (https://www.uniprot.org/, accessed on 1 March 2019). Small local alignments were removed applying some homemade filters. Sequences without a hit were annotated using BLAST V.2.2.30+ [63] against the NT database (non-redundant nucleotide sequences from all traditional divisions of GenBank, EMBL, and DDBJ excluding GSS, STS, PAT, EST, HTG, and WGS) from the National Center for Biotechnology Information (NCBI). Again, all small local alignments were removed. Identified genes were functionally annotated using the functional annotation of Uniprot [64] database for the three main functional categories (biological process, molecular function, and cellular component) with associated KEGG Ontology pathways [65] and gene Ontology database [66]. Lastly, PFam terms were obtained [67], and genome sequence was deposited at Genbank under the accession ID SAMN13178684.

### 2.4. Phylogenetic Analysis of Pseudomonas sp. PICF6

A multi-locus sequence analyses (MLSA) was conducted using the partial sequences of the housekeeping genes *gyrB* (493 nt) and *rpoD* (594 nt) to reassess the taxonomical position of *Pseudomonas* sp. PICF6, originally identified as *Pseudomonas fluorescens* PICF6 [39]. Alignments for each gene were carried out separately using Clustal version 2.0.12, and the longest common fragments were included in the analysis. Then, sequences were concatenated (*gyrB*–*rpoD*) and realigned generating a 1087 nt-long composite sequence. The *gyrB* and *rpoD* sequences of strain PICF6 were obtained from the genome here sequenced and compared to the *gyrB* and *rpoD* sequences of 42 selected *Pseudomonas* spp. type strains retrieved from different public databases, (i.e., NCBI, EMBL, KEGG, etc.). A dendrogram was generated by the Neighbor-Joining method with MEGA version 7.0 software. *Pseudomonas entomophila* L48 was used as outgroup species. Bootstrap analysis of 1000 replicates was performed to evaluate the phylogenetic tree topology.

Based on the results of the previous analysis, a genome level comparison was performed among *Pseudomonas* sp. PICF6 and reference strains of the two closest species (*Pseudomonas brassicacearum* NFM421 and *Pseudomonas corrugata* RM1-1-4, NCBI data). Moreover, *P. simiae* PICF7 was included in the comparison as our reference olive rhizobacteria. Likewise, exclusive genes present in these strains were identified. Cd-hitest [68] tool was used over the ORFs obtained from each sample with a homology level of 90% [68,69]. The newly obtained clusters were annotated against Uniprot and associated with KEGG Ontology pathways [65], gene Ontology [66] and PFam [67] terms with in-house scripts. These terms were classified following the functional classification of the same ontology. The counters were plotted into a Venn diagram for the four mentioned strains using custom Python scripts (Python Software Foundation. Python Language Reference, version 2.7. Available at http://www.python.org).

### 2.5. Colonization Ability of Olive Roots by Pseudomonas sp. PICF6

An experiment was conducted to: (i) assess the olive root colonization pattern of *Pseudomonas* sp. PICF6, and (ii) compare its colonizing ability to that of the well-known olive root inhabitant *P. simiae* PICF7 [70]. The previously-available fluorescent derivative PICF7(pLRM1) [45] was used, and a PICF6 derivative also carrying plasmid pLRM1 [71], which harbors the green fluorescent protein (GFP), was constructed as described by Montes-Osuna and co-workers [42]. Fluorescence of the bacterial cells was confirmed by using a Nikon Eclipse 80i epifluorescence microscope (Nikon Instruments Europe BV, Amstelveen, The Netherlands). Three clones of the new PICF6(pLRM1) transformant were cryopreserved in glycerol at −80 °C, and one of them was used in colonization experiments. Fluorescently-tagged bacteria were grown overnight (28 °C, 180 rpm) in LB liquid medium supplemented with gentamicin (Gm) 50 mg/L. Then, bacterial cells were collected by centrifugation and resuspended in 10 mM MgSO_4_·7H_2_O for root inoculation. Bacterial cell densities of the inocula were spectrophotometrically adjusted (A_600_ nm) at 1 × 10^8^ cfu/mL, and bacterial fluorescence was confirmed by epifluorescence microscopy before olive roots inoculation.

Three-month-old olive (cv. Picual) plants (six plants per treatment) from a commercial nursery located in Córdoba province (southern Spain) were carefully uprooted from the original substrate (nursery-made; composed of peat moss, coconut fiber and Osmocote fertilizer at 1 g/L), cleaned manually and dipped for 30 min in 300 mL of a bacterial suspension (1 × 10^8^ cfu/mL). The experiment consisted of two treatments: (i) *Pseudomonas* sp. PICF6(pLRM1), and (ii) *P. simiae* PICF7(pLRM1). The root system of non-inoculated (control treatment) plants were just dipped in 10 mM MgSO_4_·7H_2_O for 30 min. After that, each plant was carefully transplanted into 9 × 9 × 10 cm polypropylene pots filled with the same potting substrate used in the nursery (Viveros Carretero, Castro del Rio, Córdoba, Spain). To each pot, 45 mL of the bacterial cell suspension (or 10 mM MgSO_4_·7H_2_O) used in the root dipping step were added. Plants were kept under controlled conditions: 60–90% relative humidity and day/night temperatures of 25–22 °C. The photoperiod was progressively increased until reaching 14-h daylight to alleviate the potential stress experienced by the plants after being subjected to the manipulation process. The colonization of olive roots was evaluated at 3, 4, 5, 10, 17 and 20 days after inoculation (DAI) using a Axioskop 2MOTmicroscope (Carl Zeiss GmbH, Jena, Germany), controlled by Carl Zeiss Laser Scanning System LSM5 PASCAL software (Carl Zeiss). Root segments (1–4 cm long) representative of the entire root system were collected and longitudinal sections of these segments (about 30 micrometers thick) were obtained using a Vibratome Series 1000plus (TAAB Laboratories Equipment, Aldermarston, UK). The Zeiss LSM Image Browser version 4.0 (Carl Zeiss) software was used for imaging and post-processing of the confocal stacks and maximum projections.

### 2.6. Tolerance of Olive Rhizobacteria to Salt Stress

To determine the salinity tolerance level of strains PICF6 and PICF7 different media amended with 60 mM of a salt solution (75%/25% NaCl/CaCl_2_), corresponding to an electrical conductivity (EC) of 6 dS/m (the saline dose used in bioassays, see below), were assayed. For that, the EC of this salt solution was confirmed with a multi-parameter Eutech PC 700 apparatus (Thermo Fisher Scientific Inc., Singapore). Water with EC values ranging from 3 to 8 dS/m may be classify as moderately saline [72,73].

*Pseudomonas* sp. PICF6 and *P. simiae* PICF7 overnight cultures (28 °C, 180 rpm) were grown in LB. Subsequently, cultures of each bacterium were diluted to OD_600_ of 0.05 in two different liquid media, fresh LB and standard succinate medium (SSM), (6 g K_2_HPO_4_, 3 g KH_2_PO_4_, 1 g (NH_4_)_2_SO_4_, 0.2 g MgSO_4_·7H_2_O and 4 g of succinic acid in 1000 mL of distilled water, pH 7.0) amended with 60 mM of the saline solution mentioned above. Bacterial cultures in LB and SSM liquid media were used as control treatments (28 °C, 180 rpm). Aliquots from LB cultures (with or without salt) were taken at 0, 3, 7, 9, 20 and 28 h, while aliquots from SSM cultures were sampled only at 0, 20 and 28 h. Serial dilutions from each sampling time-point were grown on LB plates to calculate the number of viable cells (cfu/mL). Two independent experiments were carried out.

### 2.7. Assessment of Olive Rhizobateria as Saline or Water Stress Protectants

Greenhouse experiments were carried out to examine whether two indigenous olive rhizobacteria either showing (strain PICF6) or not (strain PICF7) ACD activity could alleviate drought or salt stress (two independent experiments for each stress condition) in young olive plants. Moreover, a combination of both strains was also assayed to evaluate possible synergistic or antagonistic effects between them. Prior to bacterial inoculation, olive plants (cv. Picual, 5-month old) purchased in the same nursery mentioned above were acclimated for 2 months in a greenhouse under natural lighting and 26–21 °C temperature range. The day before bacterial inoculation, plants were transplanted into polypropylene pots (11 × 11 × 12 cm, one per pot) containing the potting substrate indicated in Section 2.5. Inocula of strains *Pseudomonas* sp. PICF6 and *P. simiae* PICF7 were prepared as described in Montes-Osuna and co-workers [42]. For each bacterial treatment, inoculation of olive plants was carried out by adding 150 mL of a bacterial suspension adjusted at 1 × 10^8^ cfu/mL in 10 mM MgSO_4_·7H_2_O. In the case of the double treatment, the bacterial cells suspension was adjusted to a final concentration of 2 × 10^8^ cfu/mL (each of the strains adjusted to 1 × 10^8^ cfu/mL). Non-bacterized plants (control) were just drenched with 150 mL of sterile 10 mM MgSO_4_·7H_2_O.

Each experiment (two for assessing protection against drought stress and another two for evaluating protection against salt stress) consisted of 45 olive plants (cv. Picual) in which five different treatments (9 plants/treatment) were considered. Regarding salt stress assays, the treatments were: (1) control plants just irrigated with distilled water (CW), (2) plants treated with salt (S) solution (CS), (3) plants inoculated with *Pseudomonas* sp. PICF6 and treated with S solution (PICF6/S), (4) plants inoculated with *P. simiae* PICF7 and treated with S solution (PICF7/S), and (5) plants co-inoculated with strains PICF6 and PICF7 and treated with S solution (PICF6+PICF7/S). Meanwhile, drought assays included the following treatments: (1) control plants solely irrigated with tap water (CW), (2) plants subjected to drought (D) stress with no water (CD), (3) plants inoculated with *Pseudomonas* sp. PICF6 without subsequent watering (PICF6/D), (4) plants inoculated with *P. simiae* PICF7 without subsequent watering (PICF7/D), and (5) plants co-inoculated with PICF6 and PICF7 without subsequent watering (PICF6+PICF7/D).

**Figure 1:**
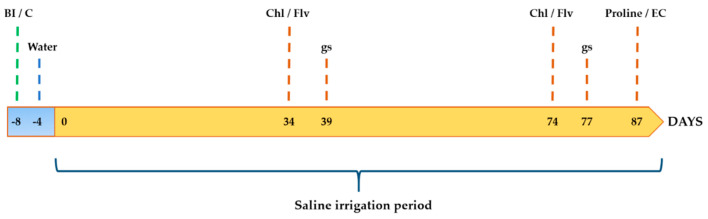
Schedule of activities performed during a salt stress experiment. Bacterial inoculation (BI) or 10 mM MgSO_4_·7H_2_O irrigation of control (C) plants is shown by a green discontinuous line (T = −8 days). Water irrigation is shown by a blue discontinuous line (T = −4 days). At time T = 0 days plants received the first dose of the salt solution. Subsequent saline doses or distilled water were applied every two or three days until the end of the experiment (87 days) (see text for details). The different physiological/biochemical parameters evaluated are shown with discontinuous brown lines. Chl, chlorophyll; EC, electrical conductivity; Flv, flavonoids; gs, stomatal conductance. This experiment was performed twice.

**Figure 2:**
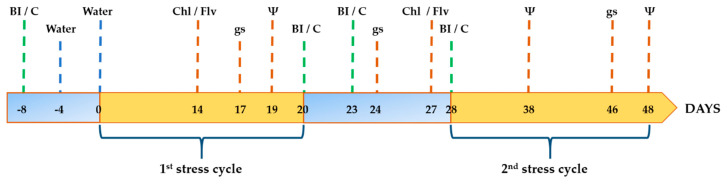
Schedule of activities performed during a drought stress experiment. The timing for bacterial inoculation (BI), or 10 mM MgSO_4_·7H_2_O amendment in the case of control (C) plants, is shown by green discontinuous lines. Water doses are shown by blue discontinuous lines. The different physiological/biochemical parameters evaluated are shown with discontinuous brown lines. Plants were subjected to two (20-days long each) drought cycles (1st and 2nd stress cycles; orange segments). Chl, chlorophyll; Flv, flavonoids; gs, stomatal conductance; Ψ, stem water potential. This experiment was performed twice.

Before (i.e., T = −8; Figure 1 and Figure 2) the onset of the drought and salt stress periods, plants of treatments 3, 4 and 5 were bacterized as previously described. Non-bacterized plants, (i.e., treatments 1 and 2), only received 150 mL of 10 mM MgSO_4_7H_2_O. Thereafter, all plants of the salt stress assays received just one dose of distilled water (100 mL) at 4 DAI (Figure 1; water irrigation). After that 100 mL of 60 mM S solution (6 dS/m; see above) were added to each pot of treatments CS, PICF6/S, PICF7/S and PICF6+PICF7/S at 8 DAI (Figure 1; 1st salt solution irrigation), while plants of treatment C were irrigated with just distilled water (100 mL). During the rest of the experiments (and up to T = 87, Figure 1) plants were irrigated (100 mL) every two-three days either with distilled water (treatment C) or S solution (rest of treatments). With regard to the drought stress assays, and after adding the first dose of bacterial cells at T = −8 (Figure 2; 1st bacterial inoculation), plants were watered twice during the first week at T = −4 and T = 0 (Figure 2). Thereafter, plants were subjected to strict drought conditions (no water supply) during 20 days (Figure 2; 1st stress cycle). At the end of this period, plants received new doses of bacteria (treatments PICF6/D, PICF7/D and PICF6+PICF7/D) or were amended with 10 mM MgSO_4_·7H_2_O (treatments CW and CD) three times during a week (Figure 2; 2nd bacterial inoculation (BI), 3rd BI and 4th BI). Subsequently, plants were subjected to a new drought cycle (Figure 2; 2nd stress cycle) during 20 days except control plants (treatment CW) that were watered as needed.

The Chl content and Flv concentration, as well as the gs (see below), were scored following the schedule showed in Figure 1 and Figure 2. Additionally, the EC of the potting substrate and the proline content in leaves and roots were determined at the end of the salt stress experiments (Figure 1). Likewise, Ψ was measured at different time-points during the drought stress experiments (Figure 2).

### 2.8. Assessment of Plant Physiological and Biochemical Parameters

Different parameters were scored to gather information about the plant physiological status during the experiments using non-destructive methods. Thus, a portable Leaf Porometer (SC-1, Decagon Devices, Pullman, WA, USA) allowed instantaneous measurements of gs in leaves. Measurements were carried out on fully-expanded and well-developed leaves (one per plant) between 12:00–16:00 h. Similarly, Chl content and Flv concentration were calculated using a Dualex 4 Scientific (FORCE-A, Orsay, Paris, France) on ten leaves per plant. Scores of these parameters were taken for all plants (9) of each of the treatments included in the experiments.

Plant water stress caused by drought conditions was monitored by measuring the Ψ at midday (13:00–14:00 h). Measurements were performed in green and well-developed leaves from the mid canopy of the plants (one per plant) that had been covered with aluminum foil at least 30 min before measurement to reduce leaf transpiration and thus equilibrate foliar and Ψ according to Abboud and co-workers [74]. Shoots were then detached and scoring of Ψ was performed using a Scholander-type pressure chamber (Model 3005F01, Santa Barbara, CA, USA).

Measurement of the proline content was performed at the end of the salt stress experiments in three plants per treatment. Leaves and roots (washed under tap water to remove potting substrate particles) were sampled and stored at −80 °C at the end of the experiments (87 days). Proline content was analyzed according to Bates and co-workers [75] with some modifications. Samples were ground in liquid nitrogen to a fine powder using a MM 301 mixer mill (Retsch GmbH, Haan, Germany). The plant tissue powder was first homogenized with 3% sulfosalicylic acid (1:10 *w*/*v*), and the homogenate was centrifuged at 2500× *g*, 4 °C during 10 min. Then, 1 mL of the extract was mixed with an equal volume of glacial acetic acid and a solution of ninhydrin 2.5% (prepared by warming in glacial acetic acid and orthophosphoric acid 85%, 60/40 [*v*/*v*]). The resulting mixtures were incubated in a glass tube for 1 h at 100 °C. Reactions were stopped by placing the tubes on ice, and 2 mL of toluene were added and mixed vigorously for 15–20 s. The upper phase was recovered and used for measuring the absorbance at 520 nm using toluene as control blank. Proline concentration was determined using L-proline to build a standard curve and calculated following the equation described by Bates and co-workers [75].

### 2.9. Electrical Conductivity (EC) of the Potting Substrate

Potting substrate samples of three different plants per treatment were collected and their EC values were scored at the end of the salt stress experiments. Plants were uprooted from the pots and the substrate contained therein was homogenized by preparing a thoroughly-mixed substrate:water (1:5 *w*/*v*) suspension. This mixture was filtered through a nylon gauze and the EC in the liquid fraction was determined by a multi-parameter Eutech PC 700 instrument.

### 2.10. Persistence of PICF6 and PICF7 Cells in Olive Roots under Drought Conditions

The survival ability of strains PICF6 and PICF7 under the drought conditions used in this study was evaluated. Olive plants (three per treatment) were inoculated with (i) PICF6(pLRM1), (ii) PICF7(pMP4655) [70] and (iii) a mixture of both strains as described in Section 2.5. Fluorescently-labelled derivatives were used to allow bacterial cell counts using selective media (Gm resistance conferred by plasmid pLRM1 and tetracycline (Tc) resistance by plasmid pMP4655). Inocula preparation and plant transplant procedure was carried out as described above. Plants were maintained in a greenhouse (under natural lighting and 26–21 °C temperature range) and subjected to drought conditions (no water supply). Stomatal conductance and Ψ measurements were used to estimate the drought stress level for each plant. When both parameters showed similar values in this set of plants to those ones scored for olive plants subjected to drought stress after 20 days (see Section 2.7), both rhizosphere/epiphytic and endophytic PICF6 and PICF7 cells were counted. To determine PICF6 and PICF7 cells present in the rhizosphere/rhizoplane, plants were carefully uprooted from the pots and 1 gr of root tissue (previously cleaned by hand) was vigorously shaken for 1 min in a 50 mL falcon tube with 20 mL of 10 mM MgSO_4_·7H_2_O containing glass beads (2 mm diameter). Subsequently, to estimate the endophytic population of the inoculated bacteria, roots were surface sterilized as described by Gómez-Lama Cabanás and co-workers [76]. Then, roots were dried on sterile filter paper and macerated using a sterilized pestle and mortar with 10 mL of 10 mM MgSO_4_·7H_2_O. Epiphytic and endophytic viable cells were determined for each plant by plating serial dilutions on LB agar plates amended with the appropriate antibiotic. Bacterial colonies were checked and counted after 24 h. Moreover, to ensure that only PICF6 and PICF7 cells were counted, 20 Gm-resistant or Tc-resistant colonies from each treatment were randomly selected and analyzed by BOX-PCR fingerprinting as previously described by Montes-Osuna and co-workers [42]. Bacterial fluorescence of the selected colonies was also confirmed by observation under epifluorescence microscope.

### 2.11. Statistical Analysis

Analysis of variance (ANOVA) was performed to determine statistical differences using the ANOVA module of Statistix 10 program (NH Analytical Software, Roseville, MN, USA). Data from proline, Flv and Chl contents, gs and Ψ parameters, and counts of epiphytic and endophytic PICF6 and PICF7 cells subjected to drought and salt stress were analyzed according to a completely randomized design. Data were tested for normality, homogeneity of variances, and subjected to whiskers and graphic boxes in order to detect the outliers, which proved their suitability for the statistical analysis. When ANOVA analysis showed significant differences among treatments, means were compared according to Fisher’s protected least significant differences (LSD) test at *p* = 0.05 or Tukey honestly-significant-difference (HSD) test at *p* = 0.05. Each experiment was analyzed separately.

## 3. Results

### 3.1. Presence of ACD Activity in Selected Indigenous Olive Rhizobacteria

**Figure 3:**
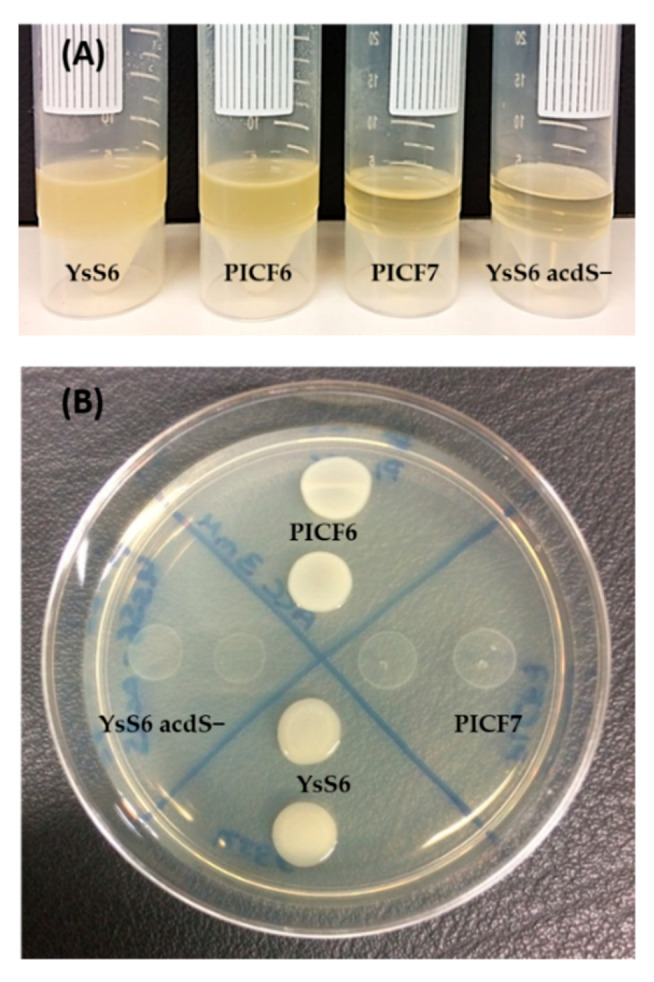
Demonstration of 1-aminocyclopropane-1-carboxylic acid (ACC) deaminase (ACD) activity in liquid (**A**) and solid (**B**) Dworkin-Foster (DF) medium using ACC as the only N source (see the Materials and Methods section for details). *Pseudomonas fluorescens* YsS6 and its ACD-defective mutant derivative YsS6 acdS- were used as positive and negative controls, respectively. PICF6, *Pseudomonas* sp. PICF6; PICF7, *Pseudomonas simiae* PICF7. The results obtained for all bacteria evaluated in this study are shown in Supplementary Table S1.

A collection of 31 indigenous olive rhizobacteria (Supplementary Table S1) were in vitro screened for the presence/absence of ACD activity, including some well-characterized BCA against *V. dahliae.* Only one strain, *Pseudomonas* sp. PICF6, was able to grow on solid and liquid DF medium supplemented with ACC (Figure 3A,B). Unexpectedly, *P. simiae* PICF7 showed no ACD activity despite a putative ACD-coding gene that was earlier predicted in its genome [41]. Similarly, *P. simiae* WCS417, nearly isogenic with strain PICF7 [42], showed no ACD activity. As expected, *P. fluorescens* YsS6 (positive control) was able to grow in DF medium amended with ACC, while its ACD-defective mutant derivative, *P. fluorescens* YsS6 acdS-, was unable to grow under these conditions.

### 3.2. Presence of an ACD-Coding Gene in Pseudomonas sp. PICF6

In order to verify the presence/absence of a true ACD-coding genes in the genome of *P. simiae* PICF7 explaining the previous result, DNA sequence comparison analysis with *acdS* (coding for ACD) genes and other closely-related deaminases (e.g., d-cysteine desulfhydrase) from different (micro)organisms were performed. *Pseudomonas* sp. PICF6 genes putatively coding for ACD and D-cysteine desulfhydrase and annotated after sequencing the genome (see below) of this olive rhizobacteria, were also included in the analysis.

**Figure 4:**
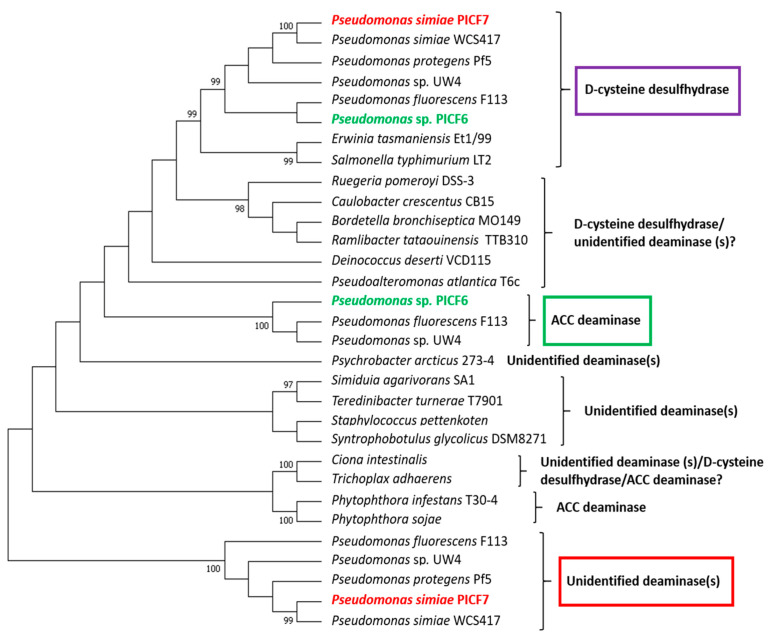
Dendrogram based on the comparison among 1-aminocyclopropane-1-carboxylic acid (ACC) deaminase, D-cysteine desulfhydrase and other unidentified deaminases coding genes from different (micro)organisms. *Pseudomonas* sp. PICF6 (green text) harbors a true ACD (green rectangle). *Pseudomonas simiae* PICF7 (red text) harbors an unidentified deaminase (red rectangle) and a d-cysteine desulfhydrase (purple rectangle), but does not harbor a true ACD. A D-cysteine desulfhydrases was also found in strain PICF6. The tree was inferred by using the Maximum Likelihood method. Only bootstrap values ≥95% based on 1000 re-sampled datasets are displayed in the phylogram branches.

Results showed that the previously predicted ACD-coding gene of *P. simiae* PICF7 actually clustered with unidentified deaminases (Figure 4, red rectangle). Moreover, this unidentified deaminase-coding gene was also present in the genome of the closely-related strain WCS417, which also yielded a negative result in the ACD test (Supplementary Table S1). This corroborates that strain PICF7 does not harbor a true ACD-coding gene. In contrast, and in agreement with the results from the ACD activity tests, *Pseudomonas* sp. PICF6 does harbor an *acdS* gene that grouped with ACD-coding genes present in *Pseudomonas fluorescens* F113 and *Pseudomonas* sp. UW4 (Figure 4, green rectangle), both bacteria displaying ACD activity [77,78]. Finally, the presence of a gene coding for a putative D-cysteine desulfhydrase was also detected in strains PICF6 and PICF7 (Figure 4, purple rectangle).

### 3.3. General Features of the Pseudomonas sp. PICF6 Genome

The genome of *Pseudomonas* sp. PICF6 consisted of a circular chromosome of 5,874,338 base pairs (bp) with an average G + C content of 60.4% (Table 1). General features of the sequencing project are shown in Supplementary Table S2. A total of 3975 protein-coding genes with function prediction were identify and listed in Supplementary Table S3. The major Clusters of Orthologous Groups (COG) categories were amino acid transport and metabolism (11.22%), signal transduction and mechanisms (8.77%), general function and prediction only (7.86%), and energy production and conversion (7.32%). Additional genome characteristics are summarized in Table 1.

### 3.4. Phylogenetic Analyses of Pseudomonas sp. PICF6 Strain

**Figure 5:**
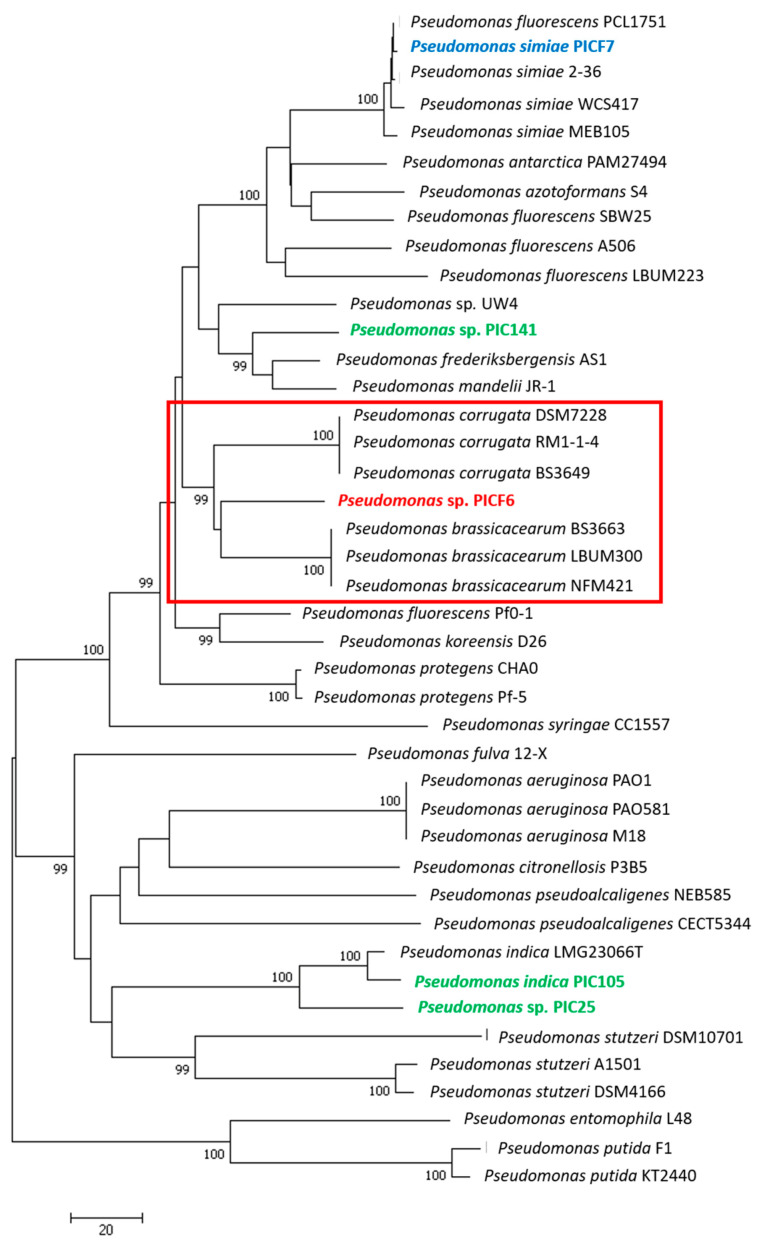
Neighbor-Joining phylogenetic tree showing the taxonomic position of *Pseudomonas* sp. PICF6 (red text) based on the alignment of concatenated partial sequences of the housekeeping genes *gyrB* and *rpoD* genes (see text for details). Red rectangles highlight the two closest species to strain PICF6. Other olive rhizobacteria included in the analysis are indicated in blue (*Pseudomonas simiae* PICF7) or green (*Pseudomonas indica* PIC105 and *Pseudomonas* spp. 25 and 141) (Supplementary Table S1). Bar indicates sequence divergence (nt). Only bootstrap values ≥95%, based on 1000 re-sampled datasets, are shown at branch nodes.

**Figure 6:**
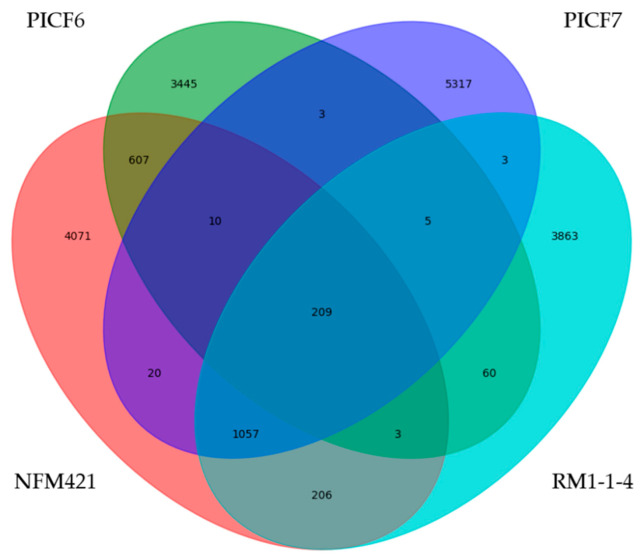
Venn diagram showing the comparison of the full sets of orthologous coding sequences present in the genomes of *Pseudomonas* sp. PICF6, *Pseudomonas brassicacearum* NFM421, *Pseudomonas corrugata* RM1-1-4 and *Pseudomonas simiae* PICF7. Figures refer to the number of orthologous coding sequences shared or not among the four strains included in the analysis.

Strain PICF6 was earlier identified as *Pseudomonas fluorescens* based on different morphological and physiological traits [39]. In the present study we aimed to reassess the identity of strain PICF6 within the *Pseudomonas* genus. Firstly, the concatenated partial sequences of two housekeeping genes (*gyrB* and *rpoD*) of different *Pseudomonas* spp. were compared. This approach allowed us to identify *P. brassicacearum* and *P. corrugata* strains as the closest relatives of strain PICF6 (Figure 5). Indeed, PICF6 showed 93.28% identity with the three *P. brassicacearum* strains included in the analysis, while with *P. corrugata* strains it showed 92.73% (BS3649 and DSM7228) and 92.91% (RM1-1-4) sequence identity. Therefore, this analysis did not allow the accurate identification of strain PICF6 at the species level. Secondly, a comparative genomics approach was carried out. Thus, the whole set of orthologous coding sequences of *Pseudomonas* sp. PICF6 was compared with those ones of two representative strains of *P. brassicacearum* and *P. corrugata* (Figure 6). Additionally, strain PICF7 was included in the analysis for comparison purposes because: (i) it is a well-characterized, beneficial olive rhizobacteria, (ii) it can be considered as a distant relative of PICF6 (Figure 5), (iii) it does not display ACD activity in contrast to PICF6, and (iv) it will be used in further experiments in this study (see below). Results showed that only 209 predicted protein coding genes were shared among the four strains, and 3445 genes were only present in the *Pseudomonas* sp. PICF6 genome. Furthermore, *Pseudomonas* sp. PICF6 and *P. brassicacearum* NFM421 only shared 829 putative protein-coding genes, while this number decrease until 277 and 227 genes when PICF6 was compared with *P. corrugata* RM1-1-4 and *P. simiae* PICF7, respectively. Whilst *Pseudomonas* sp. PICF6 shared the largest number of genes with *P. brassicacearum* NFM421, the analysis was not conclusive enough to claim PICF6 as belonging to *P*. *brassicacearum* species. Therefore, strain PICF6 remains as *incertae sedis* within the *Pseudomonas* genus.

### 3.5. Pseudomonas sp. PICF6 Is Able to Colonize the Interior of Olive Roots

**Figure 7:**
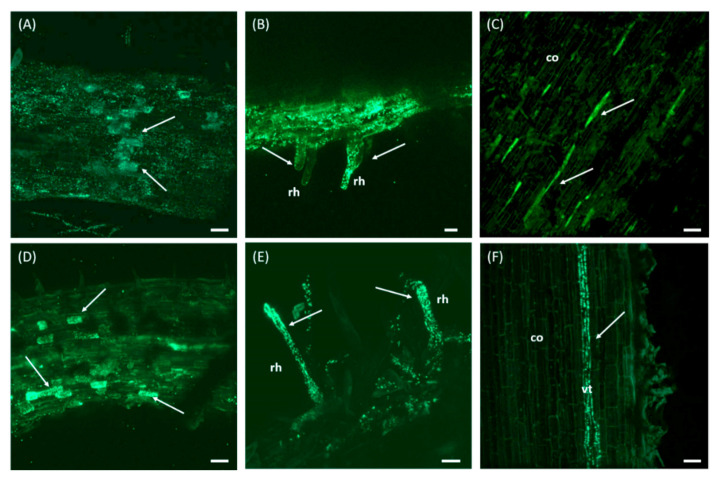
Confocal laser scanning microscopy images showing GFP-labeled *Pseudomonas* sp. PICF6 (**A**–**C**) and *Pseudomonas simiae* PICF7 (D to F) cells colonizing olive roots (cv. Picual). Panels (**A**) and (**D**) show colonization of the root surface and inner colonization of epidermal cells. Panels (**B**) and (**E**) show inner colonization of root hairs. Panels (**C**,**F**) show internal colonization of root tissues. White arrows point to spots colonized by fluorescent bacterial cells. rh, root hair; co, cortex; vt, vascular tissue. Scale bars represent 50 μm in (**A**,**C**,**D**,**F**), and 20 μm (**B**,**E**).

Confocal laser scanning microscopy (CLSM) was used to examine the olive roots colonization process of strain PICF6 and to compare it with that of the well-known BCA PICF7, which has been extensively described [42,79]. The surface of olive roots (cv. Picual) was efficiently colonized by *gfp*-tagged PICF6 (Figure 7A) and PICF7 (Figure 7D) cells, with no differences between the two strains. Moreover, the typical events of inner colonization of root hairs frequently described for PICF7 [42,79] were also detected for PICF6 cells at 3 DAI for the latter and at 5 DAI for the former (Figure 7B,D). PICF6 and PICF7 cells were eventually localized in the root cortex (Figure 7C,F) and within epidermal cells (Figure 7A,D). No fluorescent bacterial cells were detected in control (non-bacterized) plants at any time.

### 3.6. Effects of the Treatment of Biocontrol Rhizobacteria on Olive Plants Subjected to Water Stress

The overall appearance of ‘Picual’ plants at the end of the water stress experiments was similar regardless of whether or not they were treated with PICF6 (ACD positive), PICF7 (ACD negative) or a combination of both strains. Indeed, at the end of the experiments (i.e., after two cycles of 20 days under no irrigation; see Section 2.7 and Figure 2) both the canopy of the plants and their root systems did not show any significant visual difference among treatments, and all plants subjected to water stress showed less aboveground growth (as well as symptoms of wilting) compared to the control (irrigated) plants (Supplementary Figure S1).

Regarding physiological and biochemical parameters some differences were observed. For instance, the content of total Flv did not differ between CW and CD plants at 14 days from the start of the first drought cycle in any of the two experiments, indicating that drought conditions did not affect this parameter (Table 2). However, at this time point, the presence of the strains here tested, particularly PICF6 (ACD positive), induced a significant increase of the Flv content compared to non-bacterized plants, irrespective of they were irrigated or not. However, this increase was not observed in plants co-inoculated with both rhizobacteria. Interestingly, when drought stress conditions were interrupted and plants received either water (CW and CD treatments) or new doses of bacteria (PICF7/D, PICF6/D or PICF6+PICF7/D treatments), CD plants showed significantly higher Flv content compared to CW plants (Table 2) at 27 days from the start of the experiments. Moreover, plants inoculated with rhizobacteria showed a trend to enhance the total Flv content, although results differed between experiments and were less consistent than at 14 days (Table 2). This result indicated that despite the interruption of drought conditions, plants were not able to restore the Flv content scored for non-stressed plants.

Concerning the Chl content, the overall picture is that plants subjected to water stress showed higher values than the CW treatment. Moreover, treatment with the olive rhizobacteria did not produce significant effects on this parameter compared to CD plants.

**Figure 8:**
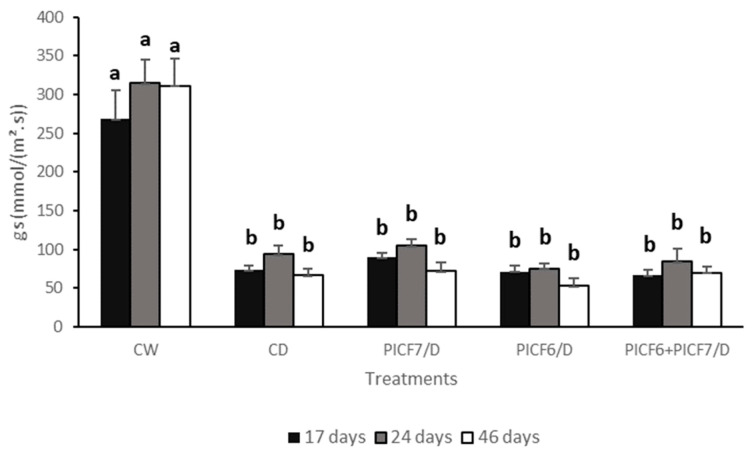
Stomatal conductance (gs) in olive plants (cv. Picual) leaves subjected to drought stress. CW, control plants solely irrigated with water; CD, plants subjected to drought stress with no water; PICF7/D, plants inoculated with *Pseudomonas simiae* PICF7 without subsequent watering; PICF6/D, plants inoculated with *Pseudomonas* sp. PICF6 without subsequent watering; PICF6+PICF7/D, plants co-inoculated with PICF6 and PICF7 without subsequent watering. Error bars represent the standard error of the means (*n* = 9). Letters represents significantly differences among treatments at the same sampling time according to Tukey (HDS) test (*p* = 0.05). This experiment was performed twice with similar results.

With regard to the gs parameter, all plants subjected to water stress showed significantly (*p* < 0.05) lower values compared to CW plants. No differences were found at any time point (17, 24 and 46 days; Figure 2), regardless of whether or not plants were treated with the olive rhizobacteria (Figure 8). Thus, treatment with PICF6, PICF7 or both strains did not modify the gs of plants subjected to water stress.

Regarding Ψ, significant differences were found at the end of each drought cycle (20 and 48 days; Figure 2) between the CW treatment and all the treatments subjected to water stress, regardless of whether or not plants were treated with the olive rhizobacteria, although some differences could be detected between experiments (Table 3).

Finally, the persistence of PICF6 and PICF7 cells on/in roots of plants subjected to drought conditions was assessed. Bacterial counts were performed when gs and Ψ reached comparable values to those scored in drought assays in order to ensure a maximum of stress conditions (see Section 2.10). Results showed that both strains were able to persist both endophytically and epiphytically, displaying similar values among treatments (Supplementary Figure S2).

### 3.7. Effects of the Treatment of Biocontrol Rhizobacteria on Olive Plants Subjected to Salt Stress

The overall appearance of the olive plants at the end of the salinity assays was similar regardless of whether or not they were treated with the tested rhizobacteria. Interestingly, most of the plants subjected to salt stress showed paraheliotropism and yellowing of leaves (Supplementary Figure S3). Thus, application of PICF6 (ACD positive), PICF7 (ACD negative) or both strains did not produce any advantage to plants subjected to salt stress, at least under our study conditions.

Similarly to plants subjected to water stress some differences were scored for physiological parameters. Thus, the total Flv content enhanced as the number of salt doses increased (Table 4). In experiment I, the presence of inoculated rhizobacteria induced a significant increase of the Flv content compared to non-bacterized plants after 34 and 74 days of continued saline irrigation, regardless of whether or not they were treated with S solution (Table 4). Nevertheless, in experiment II, a significant increase of the Flv content was detected in all plants subjected to salt stress, irrespective of they were bacterized or not (Table 4).

Regarding the Chl content variable results were obtained, particularly in experiment I (Table 4). After 34 days of continuous saline irrigation, Chl content was significantly higher in all plants subjected to salt stress compared to CW (experiment I). Although a similar trend was observed in experiment II, treatments did not show significant differences (Table 4). However, at 74 days this pattern reversed, especially in Experiment II, and the presence of the rhizobacteria inducing an overall significant decrease in Chl content compared to non-bacterized plants (Table 4).

**Figure 9:**
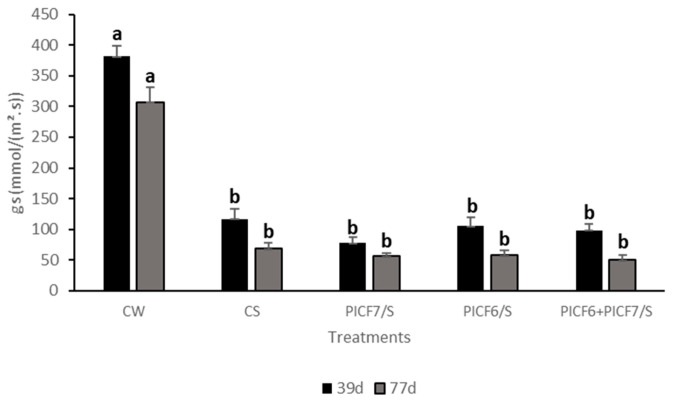
Stomatal conductance (gs) in olive plants cv. Picual. CW, control plants just irrigated with distilled water; CS, plants treated with salt (S) solution; PICF7/S, plants inoculated with *Pseudomonas simiae* PICF7 and treated with S solution; PICF6/S, plants inoculated with *Pseudomonas* sp. PICF6 and treated with S solution; PICF6+PICF7/S plants co-inoculated with strains PICF6 and PICF7 and treated with S solution. Error bars represent the standard error of the means (*n* = 9). Letters represent significantly (*p* < 0.05) differences among treatments at the same sampling time according to Tukey (HDS) test. Experiment was performed twice with similar results.

Concerning the gs parameter, all plants irrigated with S solution, independently they were previously inoculated with the rhizobacteria or not, showed significantly (*p* < 0.05) lower values compared to CW plants (Figure 9). Thus, presence of PICF6, PICF7 or both strains did not significantly protect olive plants against salt stress, at least under our experimental conditions.

All salt treatments significantly increased EC values of the potting substrate to roughly 6 dS/m at the end of the experiment, with non-significant differences among them (Table 5). Thus, a continuous input of saline water produced an accumulative effect thereby increasing EC values along time. In both experiments, EC values scored for the control treatment (CW) remained below 2 dS/m, which corresponds to non-saline soils according to classification of Dahnke and Whitney [80]. Both PICF6 and PICF7 cells fully tolerated the saline concentration eventually reached in these experiments (6 dS/m), as demonstrated by culturing experiments with LB and SSM amended with 60 mM of S solution (data not shown). Finally, and regarding the proline content in leaves and roots, results showed no significant differences among treatments (Table 5).

## 4. Discussion

Olive cultivars have traditionally been classified into three categories regarding their level of tolerance to saline soils: tolerant, moderately tolerant and sensitive [72,81]. In addition, olive trees are known for being well adapted to drought conditions usually found in the Mediterranean Basin [10,82]. The exposure of plants to salinity induces a toxic accumulation of sodium ions in plant tissues leading to plant cell damage [83]. Additionally, the accumulation of salt results in a water deficit comparable to that induced by drought. In order to alleviate the negative effects produced by salinity and/or drought, plants have developed a range of responses (see Introduction section; [84]).

In addition to plant’s defense mechanisms, the use of beneficial microorganisms can help to enhance or trigger these responses thereby reducing the negative effects caused by stress [85,86]. Related to this, the activity of the enzyme ACD, synthetized by some PGPR, has been shown to play an important role in increasing plants’ tolerance to adverse circumstances [87]. ACD decreases plant-produced ET levels under a range of abiotic stresses, which in high concentrations can lead to inhibition of plant growth or even the death of the plant [88,89]. Therefore, the use of ACD-producing PGPR to alleviate the consequences of plant exposure to stresses such as salt or drought has gained attention [90]. Despite ACD activity has been reported to be relatively common among soil borne bacteria [88], only one olive rhizobacteria (*Pseudomonas* sp. PICF6) out of the 31 here analyzed (*ad hoc* selected due to their potential/effectiveness as PGPR or BCA) showed this activity. Furthermore, absence of true ACD activity in the model BCA *P. simiae* PICF7 was somehow unexpected since a putative *acdS* gene was earlier predicted in its genome [41]. It is worth mentioning that searches in genome databases usually yield a relatively large number of closely-related *acdS* genes, but only a small fraction of them actually codes for true ACD [88]. This could be explained because many genes originally identified as putative *acdS* really encode for D-cysteine desulfhydrases [54]. In our study, we have demonstrated that *P. simiae* PICF7 harbors an unidentified deaminase and a D-cysteine desulfhydrase (Figure 4). Noteworthy, the same outcome was found for strain WCS417, a BCA nearly isogenic with PICF7 [42] that did not display ACD activity either (Supplementary Table S1). Interestingly, *Pseudomonas* sp. UW4 [91], an ACD-producing bacterium [77] included in our analysis, harbors a D-cysteine desulfhydrase and two types of deaminases, including a true ACD-coding gene that clustered with the putative *acdS* gene of *Pseudomonas* sp. PICF6 and an unidentified deaminase which is also present in *P. simiae* PICF7 (Figure 4). Strain PICF6 was earlier reported as an effective BCA against VWO affiliated to the *P. fluorescens* species [39]. However, results from our study, even at the comparative genomics level, kept this strain as *incerta sedis*, *P. corrugata* and *P. brassicacearum* being the closest relatives within the *corrugata* subgroup [92].

Results from this present study indicate that pretreatment of nursery-produced olive plants (‘Picual’) either with ACD-producing (PICF6) or ACD defective (PICF7) rhizobacteria, or with a combination of both of them, do not alleviate visual symptoms caused by drought or salt stress. However, differential physiological effects in the bacterized plants were observed. Lack of effectiveness cannot be attributed to poor colonization and persistence in/on olive roots since both strains showed the same ability to colonize and endure under the experimental conditions used. Moreover, our results demonstrate that strain PICF6 is able to endophytically colonize the olive root interior, displaying the same colonization pattern previously described for strain PICF7 [42,79]. Since strains PICF6 and PICF7 were able to tolerate the saline concentration reached in our experiments (6 dS/m), an EC value that was not significantly exceeded in the potting substrate, we can rule out that lack of protective effect could be due to toxic concentrations of salt for the introduced rhizobacteria.

One of the first plant defensive responses after prolonged exposure to drought and salinity is the decrease in Ψ and the closure of stomata [93,94,95]. A continuous water flow from the soil to the plant relies on the lower Ψ present in the plant. Changes in leaf water status are closely coordinated with stomatal closure to reduce plant water loss through transpiration [96]. Thus, as soon as plants subjected to drought or salinity conditions perceived water, they progressively increase their leaf Ψ [97,98] and gs [5,38,99,100]. In agreement with this, we observed that olive plants subjected to drought for 20 days were able to increase both gs (Figure 8) and Ψ (Table 3) after applying water (control) or new doses of bacterial suspensions, irrespective of the bacterial treatment. Changes in gs are a response to water deficit that causes a decline in rate of photosynthesis thereby reducing plant growth rate, root functionality and crop yield [11,101]. Most of the studies reported that Chl content decreases in plants subjected to water stress [21,102]. However, in our experiments, an increase in Chl was observed in plant subjected to stress compared to non-stressed plants (Table 2). While this is not a common phenomenon, increase of Chl in plants subjected to water stress [103] or no difference between stressed or non-stressed plants [38] have also been described. In our salt stress assays, a decrease in Chl content was observed only after 74 days of continuous application of salt solution (Table 4). That is, plants needed to be subjected to a certain and cumulative salt concentration to unleash this response [104]. However, neither plants subjected to drought nor salt stress experienced significant changes in any of the above mentioned parameters, regardless of whether or not they were treated with any of the two tested rhizobacteria. This finding was not totally unexpected. Indeed, Rolli and co-workers [37] studied the potential of several rhizobacteria from grapevine plants in alleviating drought stress symptoms. Their results showed an important variability. Moreover, many ACD-producing bacteria were not able to improve gs or water use efficiency values in comparison to non-bacterized grapevine plants subjected to drought stress. Likewise, wheat plants inoculated with strains of *Kocuria rhizophila* and *Cronobacter sakazakii* species (both ACD-producing bacteria) and subjected to saline irrigation (80 and 160 mM) showed similar Chl values than non-stressed plants [105].

Drought and salinity also induces overproduction of reactive oxygen species (ROS) in the plant causing oxidative stress in the cell. Flv are non-enzymatic antioxidant compounds produced by plants to cope with oxidative stress produced, for instance, by salt and drought stresses [32,106]. In our study, Flv content significantly increased in plants subjected to stress (salt or drought) compared to CW plants. Even though our results are in agreement with those presented by several authors, the ability of olive plants to scavenge ROS seems to be cultivar dependent [21,107]. The content of Flv was significantly higher in plants inoculated with PICF6, PICF7 or both strains and subjected to salt stress compared to control or non-bacterized plants, at least in one of the experiments performed (Table 4). This suggested that the two rhizobacteria tested triggered a protective response in olive plants against ROS produced under the stressing conditions assayed. Likewise, similar results were obtained in drought assays in which plants inoculated with strain PICF6 (and to a lesser extent with PICF7) showed significantly higher Flv values (Table 2). Kang and co-workers [108] reported higher Flv content in soybean plants subjected to drought or salinity that were previously inoculated with the ACD-producing strain *P. putida* H-2-3, compared to non-bacterized plants. Our overall results, however, did not seem to differentiate between the effects induced by ACD producing (PICF6) or ACD defective (PICF7) strains.

Finally, the accumulation of osmolytes such as proline is one of the most frequent adaptation response observed in plants to alleviate the loss of cell turgidity [94,106]. No significant differences were detected in the proline content of leaves and roots between salt-stressed and non-stressed plants at the end of our experiments. Increased proline concentration in olive leaves and roots has been earlier reported as a response to water or saline stress [109]. Nevertheless, other reports have suggested the opposite situation [38,110]. Accumulation of proline in olive tissues under stress conditions seems to be cultivar dependent. Indeed, Aparicio and co-workers [111] studied the proline content in six olive cultivars subjected to salt stress (200 mM). They observed that proline content decreased, increased or remained constant depending on the cultivar tested. More specifically, cv. Picual, the variety used in our study, showed no differences in proline content when plants were subjected to salt stress compared to control plants, a result in full agreement with our findings.

In summary, gs, Ψ, Chl and proline values did not improve upon applying the selected microorganisms. However, the Flv content was an exception, and increased values in this parameter after the addition of the two *Pseudomonas* strains was observed. Overall, under our greenhouse experimental conditions, the introduction of the tested rhizobacteria did not provide advantages to olive plants to better cope with water scarcity or salt accumulation. It cannot be ruled out that the tested olive plants harbor other endophytic bacteria already contributing to the level of tolerance they display to theses stresses. Moreover, since one of the strains (PICF6) displayed ACD activity, our results did not support any protective role of PICF6 ACD against drought or saline stress. It would be interesting to investigate whether the *acdS* gene of PICF6 is expressed in the olive root system, what might explain the same neutral outcome observed for the ACD-defective strain PICF7. Likewise, the presence of an alternate mechanism recently reported [112] involved in ET production by endophytic bacteria, and helping the host plant to adapt to stress, deserves to be investigated. However, from a practical point of view, these two BCA do not seem to pose added value as for their capacity to ameliorate the effects caused by the abiotic stresses evaluated in this study.

## Figures and Tables

**Table 1:** Genomic features, gene prediction and annotation summary of the *Pseudomonas* sp. PICF6 genome.

Attribute	Value
Total sequence length (bp)	5,874,338
Total ungapped length (bp)	5,870,039
Number of scaffolds	35
Scaffold N50	407,288
Scaffold L50	6
Number of contigs	397
Contig N50	29,701
Contig L50	64
Total genes	5270
Protein-coding genes	4961
Genes (RNA)	53
CDSs (total)	5217
tRNAs	48
ncRNAs	4
Pseudo Genes (total)	256
Protein-coding genes with function prediction	3975
Protein-coding genes assigned to COGs	2936
CRISPR repeats	3

**Table 2:** Measurements of total flavonoids (Flv) and chlorophyll (Chl) in olive (cv. Picual) leaves subjected to drought stress.

	**Experiment I**			
	**Flv Content**		**Chl Content**	
	**14 days**	**27 days**	**14 days**	**27 days**
**Treatments**				
CW	0.84**c**	0.84**c**	48.42**b**	44.95**b**
CD	0.88**bc**	0.98**ab**	54.93**a**	52.67**a**
PICF7/D	0.90**b**	0.97**b**	55.88**a**	53.79**a**
PICF6/D	0.98**a**	1.03**a**	51.13**b**	50.72**a**
PICF6+PICF7/D	0.90**b**	1.02**ab**	54.14**a**	52.13**a**
	**Experiment II**			
CW	0.81**b**	0.80**d**	53.43**a**	51.29**c**
CD	0.84**b**	0.88**bc**	54.21**a**	54.85**a**
PICF7/D	0.93**a**	0.99**a**	53.06**a**	54.64**a**
PICF6/D	0.91**a**	0.95**ab**	53.10**a**	53.82**ab**
PICF6+PICF7/D	0.82**b**	0.85**cd**	51.12**a**	51.47**bc**

Values are expressed in Dualex units. CW, control plants solely irrigated with water; CD, plants subjected to drought stress with no water; PICF7/D, plants inoculated with *Pseudomonas simiae* PICF7 without subsequent watering; PICF6/D, plants inoculated with *Pseudomonas* sp. PICF6 without subsequent watering; PICF6+PICF7/D, plants co-inoculated with PICF6 and PICF7 without subsequent watering. Data are means of ten leaves per plant (nine plants per treatment). Different letters in a column indicate significant differences among treatments at the same sampling time according to Fisher’s protected LSD test (*p* < 0.05).

**Table 3:** Stem water potential (Ψ) values of olive plants subjected to drought stress.

**Treatments**	**Experiment I**		
	**19 Days**	**38 Days**	**48 Days**
CW	−10.00**b**	−9.67**b**	−10.50**b**
CD	−55.33**a**	−14.83**a**	−61.33**a**
PICF7/D	−64.67**a**	−18.17**a**	−47.50**a**
PICF6/D	−66.17**a**	−14.83**a**	−48.83**a**
PICF6+PICF7/D	−66.33**a**	−17.50**a**	−55.50**a**
	**Experiment II**		
CW	−8.33**c**	−11.17**a**	−10.17**b**
CD	−53.17**b**	−11.67**a**	−53.67**a**
PICF7/D	−64.67**a**	−10.67**a**	−72.33**a**
PICF6/D	−56.50**ab**	−11.83**a**	−64.33**a**
PICF6+PICF7/D	−55.00**ab**	−11.33**a**	−47.67**a**

Values are expressed in MPa. Data are means of three plants. Different letters in a column represent significant differences among treatments at the same sampling time according to Tukey (HDS) test. CW, control plants solely irrigated with water; CD, plants subjected to drought stress with no water; PICF7/D, plants inoculated with *Pseudomonas simiae* PICF7 without subsequent watering; PICF6/D, plants inoculated with *Pseudomonas* sp. PICF6 without subsequent watering; PICF6+PICF7/D, plants co-inoculated with PICF6 and PICF7 without subsequent watering.

**Table 4:** Measurement of total flavonoids (Flv) and chlorophyll (Chl) in olive (cv. Picual) leaves subjected to salt stress.

**Treatments**	**Experiment I**			
	**Flv Content**		**Chl Content**	
	**34 Days**	**74 Days**	**34 Days**	**74 Days**
CW	0.85**b**	0.88**c**	47.15**c**	49.83**a**
CS	0.89**b**	1.09**b**	51.48**ab**	44.86**b**
PICF7/S	0.97**a**	1.18**a**	51.91**a**	46.84**ab**
PICF6/S	0.98**a**	1.18**a**	49.81**b**	40.43**c**
PICF6+PICF7/S	0.95**a**	1.17**a**	51.56**ab**	47.93**ab**
	**Experiment II**			
CW	0.80**b**	0.82**b**	51.00**a**	53.82**a**
CS	0.93**a**	1.11**a**	55.08**a**	52.10**a**
PICF7/S	0.95**a**	1.06**a**	53.86**a**	45.35**b**
PICF6/S	0.96**a**	1.06**a**	52.80**a**	47.67**b**
PICF6+PICF7/S	1.00**a**	1.11**a**	52.13**a**	47.14**b**

Values are expressed in Dualex units. CW, control plants just irrigated with distilled water; CS, plants treated with salt (S) solution; PICF7/S, plants inoculated with *Pseudomonas simiae* PICF7 and treated with S solution; PICF6/S, plants inoculated with *Pseudomonas* sp. PICF6 and treated with S solution; PICF6+PICF7/S plants co-inoculated with strains PICF6 and PICF7 and treated with S solution. Data are means on ten leaves per each plant (nine plants per treatments). Different letters indicate significant differences among treatments at the same sampling time according to Fisher’s protected LSD test (*p* < 0.05).

**Table 5:** Measurements of electrical conductivity (EC) and proline content in leaves and roots of ‘Picual’ plants at the end of the salt stress experiment.

Treatments	EC	Proline Content (Leaves)	Proline Content (Roots)
CW	1.32**b**	6.80**a**	1.33**a**
CD	6.24**a**	6.58**a**	1.68**a**
PICF7/D	5.73**a**	9.02**a**	1.94**a**
PICF6/D	6.00**a**	9.89**a**	1.46**a**
PICF6+PICF7/D	7.00**a**	8.70**a**	1.85**a**

Units of EC are expressed in dS/m. Proline content (leaves and roots) is expressed in µmol proline/g of fresh weight material. CW, control plants just irrigated with distilled water; CS, plants treated with salt (S) solution; PICF7/S, plants inoculated with *Pseudomonas simiae* PICF7 and treated with S solution; PICF6/S, plants inoculated with *Pseudomonas* sp. PICF6 and treated with S solution; PICF6+PICF7/S plants co-inoculated with strains PICF6 and PICF7 and treated with S solution. Data are means on three plants. Within each column, different letters indicate significant (*p* < 0.05) differences among treatments according to Tukey (HDS) test. EC and proline measurements were performed for the two experiments obtaining similar results.

## Data Availability

The genome sequence of *Pseudomonas* sp. PICF6 is deposited at NCBI under the BioProject ID PRJNA587116, BioSample ID SAMN13178684.

## Supplementary Materials

The following are available online at https://www.mdpi.com/article/10.3390/microorganisms9061209/s1, Table S1. Bacterial strains screened for the presence of 1-aminocyclopropane-1-carboxylic acid deaminase (ACD) activity, Table S2. General information of the *Pseudomonas* sp. PICF6 sequencing project, Table S3. Number of genes associated with general COG functional categories, Figure S1. Overall appearance of representative olive plants (upper image) cultivar Picual and roots (bottom images) subjected to drought stress, Figure S2. Endophytic and epiphytic populations of *Pseudomonas simiae* PICF7 and *Pseudomonas* sp. PICF6 in/on olive roots of ‘Picual’ plants subjected to water stress, Figure S3. Overall appearance of olive plants cv. Picual subjected to salt stress.
