# Supplementary material for: Evaluation of Indigenous Olive Biocontrol Rhizobacteria as Protectants against Drought and Salt Stress
Source: Microorganisms. 2021 Jun 3;9(6):1209. doi: 10.3390/microorganisms9061209 (PMC8230297; doi:10.3390/microorganisms9061209)
Supplement: Supplementary file 1 [file microorganisms-09-01209-s001.zip › Montes-Osuna et al. Microorganisms (Supplementary material) Final(Nuria).pdf]

Supplementary material

Table S1. Bacterial strains screened for the presence of 1-aminocyclopropane-1-carboxylic acid deaminase (ACD) activity.

**Bacterial strains**	**Origin**	**Characteristic (s)**	**Reference**	**ACD activity**
Agrobacterium tumefaciens PIC66	Olive root	n.a	Lab. collection	-
Bacillus sp. PIC28	Olive root	BCA	[48]	-
Bacillus sp. PIC22	Olive root	n.a	Lab. collection	-
Paenibacillus polymyxa PIC73	Olive root	BCA	[48]	-
Paenibacillus terrae PIC167	Olive root	BCA	[48]	-
Pseudomonas fluorescens YsS6	Tomato plants	PGPR	[51]	+
P. fluorescens YsS6 acdS–		*Acds*- mutant	[52]	-
Pseudomonas indica PIC2	Olive root	n.a	Lab. collection	-
P. indica PIC15	Olive root	n.a	Lab. collection	-
P. indica PIC18	Olive root	n.a	Lab. collection	-
P. indica PIC31	Olive root	n.a	Lab. collection	-
P. indica PIC37	Olive root	n.a	Lab. collection	-

P. indica PIC46	Olive root	n.a	Lab. collection	-
P. indica PIC85	Olive root	n.a	Lab. collection	-
P. indica PIC102	Olive root	n.a	Lab. collection	-
P. indica PIC105	Olive root	BCA	[47]	-
P. indica PIC128	Olive root	n.a	Lab. collection	-
P. indica PIC133	Olive root	n.a	Lab. collection	-
P. indica PIC143	Olive root	n.a	Lab. collection	-
P. indica PIC144	Olive root	n.a	Lab. collection	-
P. indica PIC148	Olive root	n.a	Lab. collection	-
P. indica PIC159	Olive root	n.a	Lab. collection	-
P. indica PIC163	Olive root	n.a	Lab. collection	-
Pseudomonas putida PICP2	Olive root	n.a	Lab. collection	-
Pseudomonas simiae PICF7	Olive root	BCA/PGPR	[41,42]	-
P. simiae WCS417	Wheat root	PGPR	[53]	-
Pseudomonas sp. PIC25	Olive root	BCA	[47]	-

Pseudomonas sp. PIC59	Olive root	n.a	Lab. collection	-
Pseudomonas sp. PIC111	Olive root	n.a	Lab. collection	-
Pseudomonas sp. PIC115	Olive root	n.a	Lab. collection	-
Pseudomonas sp. PIC141	Olive root	BCA	[47]	-
Pseudomonas sp. PICF6	Olive root	BCA	[39]	+
Sphingomonas sp. PIC121	Olive root	n.a	Lab. collection	-
Streptomyces sp. PIC71	Olive root	n.a	Lab. collection	-

BCA, biological control agent; Lab. Collection, culture collection of the Laboratory of Plant-Microorganism Interactions; n.a, not available; PGPR, plant growth promoting rhizobacteria

Table S2. General information of the *Pseudomonas* sp. PICF6 sequencing project.

MIGS ID	Property	Term
MIGS-28	Libraries used	PCR-free 550 pb
MIGS-29	Sequencing platforms	Illumina MiSeq
MIGS-30	Assemblers	Megahit v. 1. 1. 2
MIGS-31	Finishing quality	Finished
MIGS-31.2	Fold coverage	150 x
MIGS-32	Gene calling method	NCBI Prokaryotic Genome Annotation Pipeline (PGAP)
	Locus Tag	PICF6
	Genbank ID	WUUD00000000
	BioProject	PRJNA587116
	NCBI taxon ID	286
	Project relevance	Plant-bacteria interaction, Model for endophytic lifestyle, Agricultural, Environmental

MIGS, minimum information about a genome sequence

Table S3. Number of genes associated with general COG functional categories

Code	Value	% of total*	Description
A	1	0,028	RNA processing and modification
B	0	0,000	Chromatin structure and dynamics
C	257	7,318	Energy production and conversion
D	34	0,968	Cell cycle control, cell division, chromosome partitioning
E	394	11,219	Amino acid transport and metabolism
F	86	2,449	Nucleotide transport and metabolism
G	166	4,727	Carbohydrate transport and metabolism
H	197	5,609	Coenzyme transport and metabolism
I	157	4,470	Lipid transport and metabolism
J	230	6,549	Translation, ribosomal structure and biogenesis
K	233	6,634	Transcription
L	129	3,673	Replication, recombination and repair
M	208	5,923	Cell wall/membrane/envelope biogenesis
N	117	3,331	Cell motility
O	143	4,072	Posttranslational modification, protein turnover, chaperones
P	217	6,179	Inorganic ion transport and metabolism
Q	78	2,221	Secondary metabolites biosynthesis, transport and catabolism
R	276	7,859	General function prediction only
S	123	3,502	Function unknown
T	308	8,770	Signal transduction mechanisms
U	60	1,708	Intracellular trafficking, secretion, and vesicular transport
V	66	1,879	Defense mechanisms
W	11	0,313	Extracellular structures
X	21	0,598	Mobilome: prophages, transposons
Y	0	0,000	Nuclear structure
Z	0	0,000	Cytoskeleton

* The total is based on the total number of protein coding genes in the annotated genome

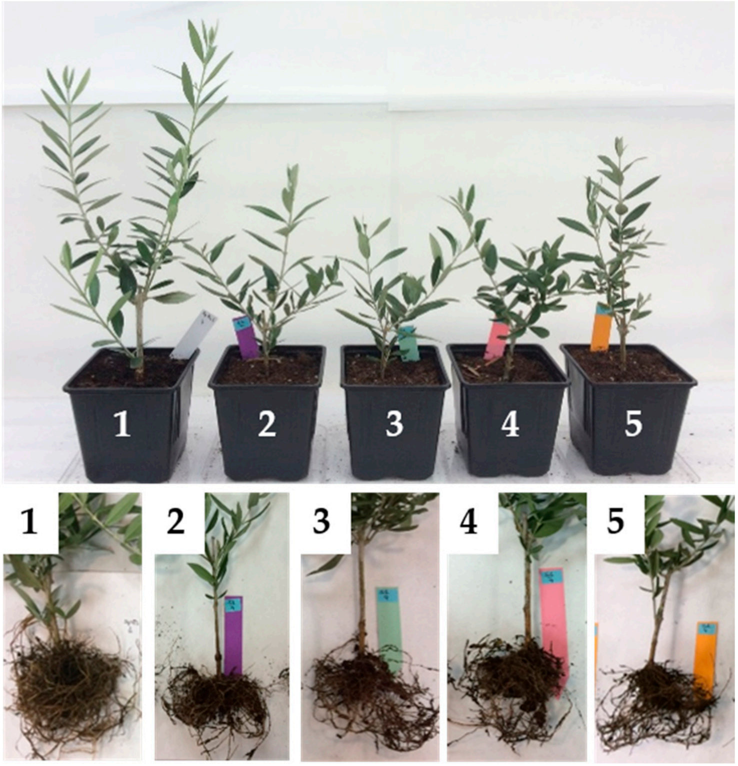

Figure S1. Overall appearance of representative olive plants (upper image) cultivar Picual and roots (bottom images) subjected to drought stress. 1, control plant solely irrigated with water; 2; plant subjected to drought stress with no bacterial treatment; 3, plant inoculated with *Pseudomonas simiae* PICF7 without subsequent watering; 4, plant inoculated with *Pseudomonas* sp. PICF6 without subsequent watering; 5, plant co-inoculated with PICF6 and PICF7 without subsequent watering.

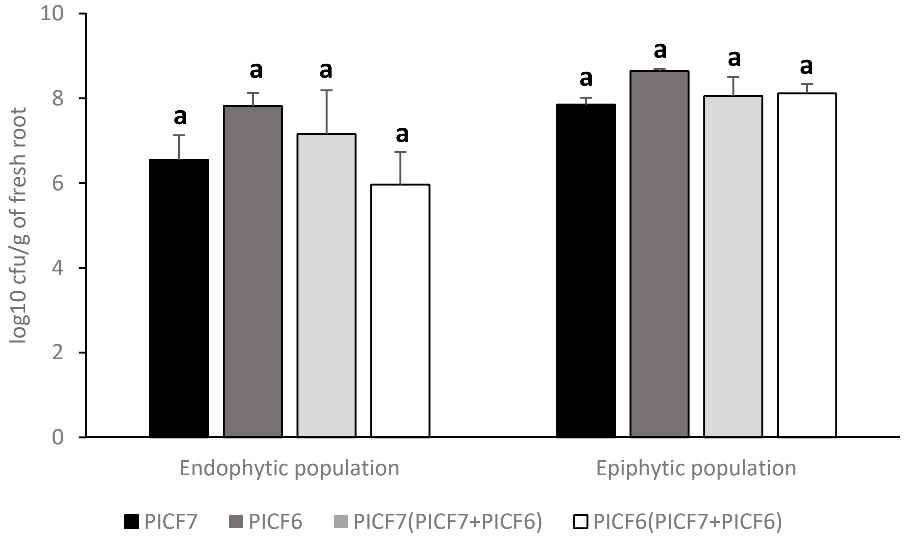

Figure S2. Endophytic and epiphytic populations of *Pseudomonas simiae* PICF7 and *Pseudomonas* sp. PICF6 in/on olive roots of 'Picual' plants subjected to water stress. Error bars represent the standard error of the means (n=3). Different letters (for each compartment) indicate significant (p < 0.05) differences among treatments according to Tukey (HDS) test. PICF7; *Pseudomonas simiae* PICF7. PICF6; *Pseudomonas* sp. PICF6. PICF7+PICF6 refers to the co-inoculation with both strains. PICF7 (PICF7+PICF6) and PICF6 (PICF7+PICF6) represent the counts for each bacterium in the co-inoculation treatment. This experiment was performed twice with similar results.

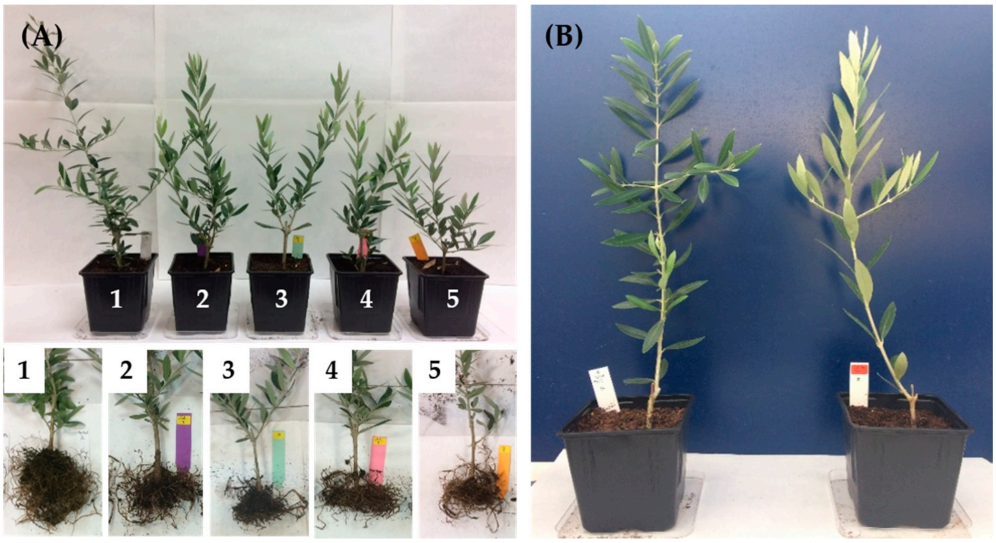

Figure S3. Overall appearance of olive plants cv. Picual subjected to salt stress. Panel (A) shows olive plants (upper image) representative of each of the treatments at the end of the experiment. Lower images show the root systems of the same plants. Panel (B) shows an olive plant just irrigated with distilled water (left) and olive plant treated with salt solution (right) showing paraheliotropism and yellowing of leaves. 1, control plant just irrigated with distilled water; 2, plant treated with salt (S) solution; 3, plant inoculated with *Pseudomonas simiae* PICF7 and treated with S solution; 4, plant inoculated with *Pseudomonas* sp. PICF6 and treated with S solution; 5 plant co-inoculated with strains PICF6 and PICF7 and treated with S solution.
