# Supplementary figures and images for: Evaluation of Indigenous Olive Biocontrol Rhizobacteria as Protectants against Drought and Salt Stress
Source: Microorganisms. 2021 Jun 3;9(6):1209. doi: 10.3390/microorganisms9061209 (PMC8230297; doi:10.3390/microorganisms9061209)

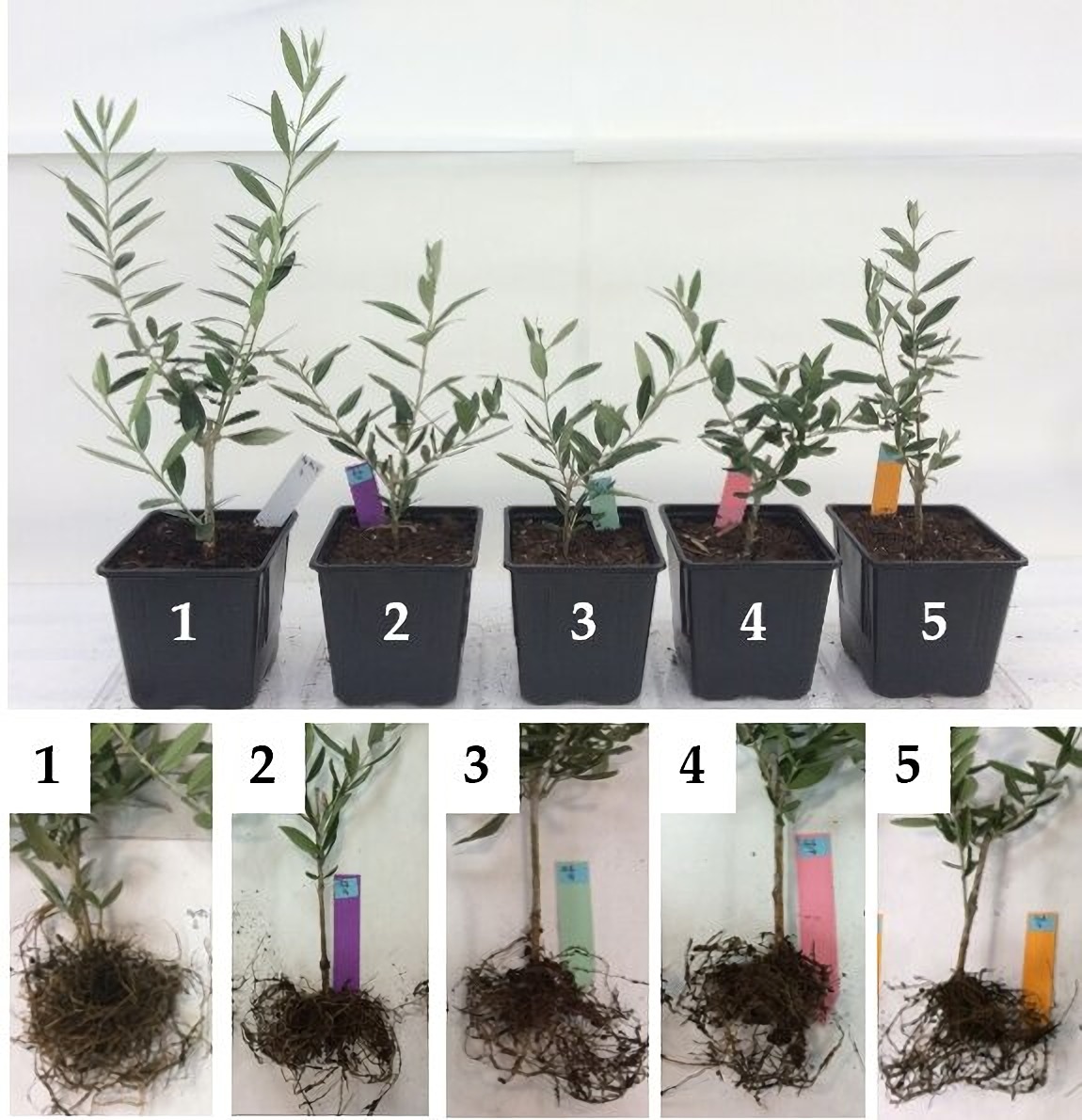

Supplement: Supplementary file 1 [file microorganisms-09-01209-s001.zip › Figure S1.jpg]

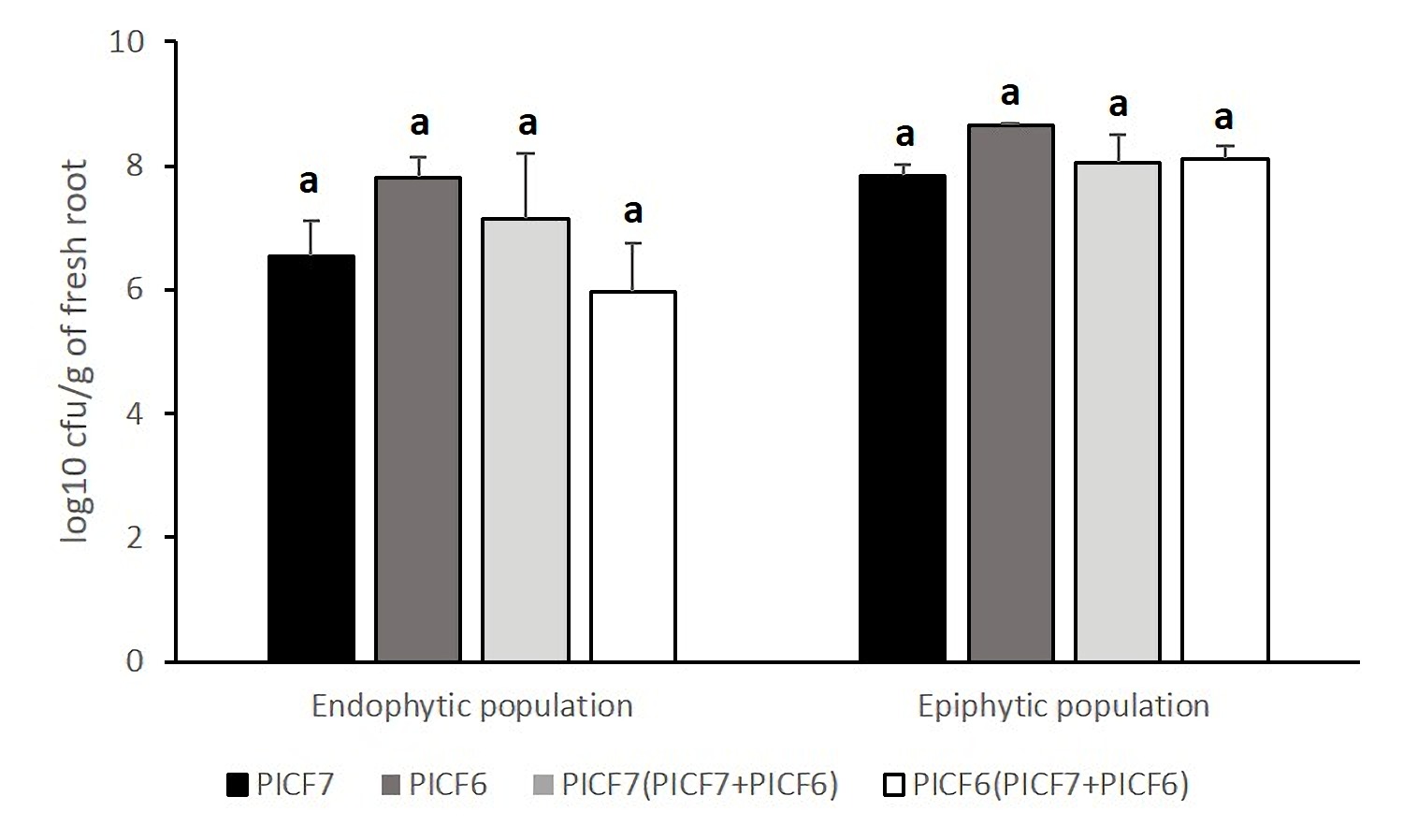

Supplement: Supplementary file 1 [file microorganisms-09-01209-s001.zip › Figure S2.jpg]

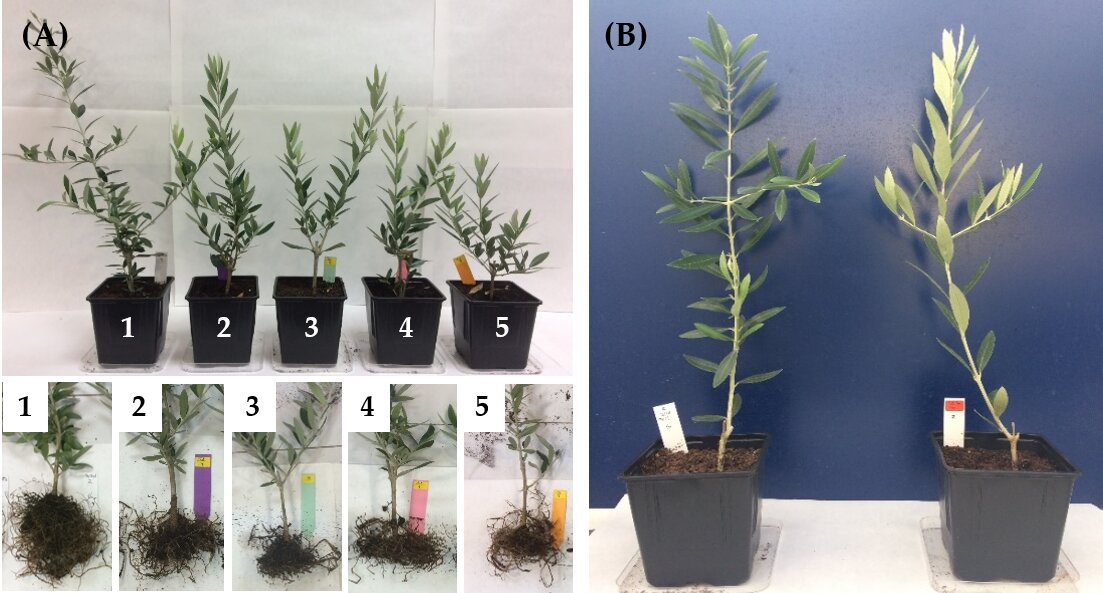

Supplement: Supplementary file 1 [file microorganisms-09-01209-s001.zip › Figure S3.jpg]
